# Preferential expression of mutant *ABCD1 *allele is common in adrenoleukodystrophy female carriers but unrelated to clinical symptoms

**DOI:** 10.1186/1750-1172-7-10

**Published:** 2012-01-26

**Authors:** Ettore Salsano, Silvia Tabano, Silvia M Sirchia, Patrizia Colapietro, Barbara Castellotti, Cinzia Gellera, Marco Rimoldi, Viviana Pensato, Caterina Mariotti, Davide Pareyson, Monica Miozzo, Graziella Uziel

**Affiliations:** 1 Department of Clinical Neurosciences, Fondazione IRCCS, Istituto Neurologico "C. Besta", Milan, Italy; 2 Department of Medicine, Surgery and Dentistry, Università degli Studi di Milano, Milan, Italy; 3 Pathology Unit, Fondazione IRCCS, Ca' Granda, Ospedale Maggiore Policlinico, Milan, Italy; 4 Department of Diagnostics and Applied Technology, Fondazione IRCCS, Istituto Neurologico "C. Besta", Milan, Italy; 5 Department of Pediatric Neurosciences, Fondazione IRCCS, Istituto Neurologico "C. Besta", Milan, Italy

**Keywords:** X-linked Adrenoleukodystrophy, X Chromosome inactivation, *ABCD1*, Allele-specific expression.

## Abstract

**Background:**

Approximately 20% of adrenoleukodystrophy (X-ALD) female carriers may develop clinical manifestations, typically consisting of progressive spastic gait, sensory deficits and bladder dysfunctions. A skewing in X Chromosome Inactivation (XCI), leading to the preferential expression of the X chromosome carrying the mutant *ABCD1 *allele, has been proposed as a mechanism influencing X-linked adrenoleukodystrophy (X-ALD) carrier phenotype, but reported data so far are conflicting.

**Methods:**

To shed light into this topic we assessed the XCI pattern in peripheral blood mononuclear cells (PBMCs) of 30 X-ALD carriers. Since a frequent problem with XCI studies is the underestimation of skewing due to an incomplete sample digestion by restriction enzymes, leading to variable results, we developed a pyrosequencing assay to identify samples completely digested, on which to perform the XCI assay. Pyrosequencing was also used to quantify *ABCD1 *allele-specific expression. Moreover, very long-chain fatty acid (VLCFA) levels were determined in the same patients.

**Results:**

We found severely (≥90:10) or moderately (≥75:25) skewed XCI in 23 out of 30 (77%) X-ALD carriers and proved that preferential XCI is mainly associated with the preferential expression of the mutant *ABCD1 *allele, irrespective of the manifestation of symptoms. The expression of mutant *ABCD1 *allele also correlates with plasma VLCFA concentrations.

**Conclusions:**

Our results indicate that preferential XCI leads to the favored expression of the mutant *ABCD1 *allele. This emerges as a general phenomenon in X-ALD carriers not related to the presence of symptoms. Our data support the postulated growth advantage of cells with the preferential expression of the mutant *ABCD1 *allele, but argue against the use of XCI pattern, *ABCD1 *allele-specific expression pattern and VLCFA plasma concentration as biomarkers to predict the development of symptoms in X-ALD carriers.

## Background

X-linked adrenoleukodystrophy (X-ALD, OMIM#300100) encompasses a spectrum of X-linked metabolic diseases due to mutations in the *ABCD1 *(*A*TP-*B*inding *C*assette, Subfamily *D*, Member 1) gene mapping to Xq28. *ABCD1 *encodes for an integral peroxisomal membrane protein which is hypothesized to be necessary for transferring very long-chain fatty acids (VLCFAs) or their activated metabolites into the peroxisomes, where they are degraded through the beta-oxidation pathway. Hence, X-ALD is biochemically characterized by excessive plasma and tissue accumulation of VLCFAs. It has been suggested that the consequence of the impairment of VLCFA beta-oxidation is an oxidative stress, provoking an axonal damage [1]. Although X-ALD is associated with mutations in the sole *ABCD1 *gene, several different phenotypes are recognized, mainly depending on age of onset, and presence and type of neurological findings. This heterogeneous spectrum comprises the development of symptoms in about 20% of female carriers, usually in their thirties [2,3]. Symptoms of female X-ALD carriers typically consist of an AMN-like phenotype (i.e. progressive spastic gait, sensory deficits and bladder dysfunction, but no adrenal insufficiency).

X Chromosome Inactivation (XCI) is the silencing of one of the two X chromosomes in mammal female cells, to ensure an equal expression of X-linked genes between the two sexes. This process takes place during early development, when, within the same nucleus, one X is transcriptionally silenced while the other remains active. Usually, the choice of which X to inactivate is random, therefore about 50% of cells express genes from the paternal-derived X chromosome, and 50% from the maternal one. Once established, the inactive state is stably maintained through cell division so that females can be considered as a "mosaic" in regards to X chromosome gene expression. Importantly, not all X-linked genes are completely silenced on the inactive X, and some of them escape inactivation, being expressed by both the active and the inactive X.

In X-linked disorders, the widely variable penetrance in female carriers can be dependent upon multiple mechanisms and can be related to the XCI pattern [4]. In a subgroup of females, XCI is preferential and results in an unbalanced expression of X-linked genes among the cells, usually favoring the expression of the wild-type allele [5,6]. The preferential expression of the mutant allele is also conceivable and could be related to the presence of symptoms.

In X-ALD female carriers, the hypothesis that the skewing of XCI, favoring the expression of the mutant *ABCD1 *allele in peripheral blood mononuclear cells (PMBCs), may be related to clinical symptoms is intriguing. However, conflicting results have been reported by different authors: Watkiss *et al. *[7] failed to find any association in 12 X-ALD female carriers, whereas Maier *et al. *[8], who analyzed 22 X-ALD female carriers, found that skewed XCI was related to neurological manifestation. Moreover, the question of whether the wild-type or the mutant allele is predominantly active in symptomatic female carriers remains mostly unanswered, since Maier *et al. *found inconsistent results in the fibroblasts of only three carriers [8].

Certainly, the demonstration of an unambiguous relation between skewed XCI in PBMCs and clinical symptoms would have important consequences on the clinical practice. Indeed, it would be a valuable parameter to early identify the subgroup of X-ALD female carriers with a higher-risk of becoming symptomatic and for whom a possible preventive therapy would be warranted [9]. With the aim of shedding light on this topic, we assessed the XCI pattern in 30 X-ALD female carriers and evaluated its relation with clinical findings. In addition, we quantitatively measured the *ABCD1 *allele-specific expression in our cohort of carriers, in order to establish whether the direction of XCI pattern favors the expression of the mutant or the wild-type allele. Finally, we investigated the correlations between VLCFA levels and the degree of expression of mutant *ABCD1 *allele in PBMCs.

## Patients and methods

### Patients

We studied 31 women from 14 families segregating *ABCD1 *mutations (Table 1). See Additional file 1, Figure S1 and Table 2 for further information.

**Table 1:** Clinical Findings, Genotype, X-Chromosome Inactivation (XCI), *ABCD1 *Allele-Specific Expression (ASE) and Biochemical Findings (VLCFA plasma levels) of X-ALD carriers

Nr of family, consultants	Age (yrs)	Presence of symptoms (age at onset, yrs)	Mutations		XCI pattern	*ABCD1 *ASE (mut:wt)	C26 (nv)	C26/C22 (nv)	C24/C22 (nv)
**F1 II-3**	67	Yes (45)	410G > A	W137X	97:03	84:16	1,09 (<0,75)	48 (<17)	1644 (<1100)

**F1 III-2**	34	No	410G > A	W137X	91:09	nd	0,58 (<0,75)	47 (<17)	1482 (<1100)

**F2 I-2**	61	Yes (59)	427C > G	P143A	71:29	93:07	0,85 (<0,75)	18 (<17)	1222 (<1100)

**F2 II-1**	38	No	427C > G	P143A	85:15	83:17	nd	nd	nd

**F2 II-2**	35	No	427C > G	P143A	76:24	77:23	nd	nd	nd

**F3 II-2**	73	Yes (45)	428C > A	P143H	60:40	38:62	1,45 (<1,50)	28 (<40)	700 (<820)

**F3 III.1**	46	No	428C > A	P143H	84:16	84:16	1,53 (<1,50)	40 (<40)	860 (<820)

**F3 III-2**	50	No	428C > A	P143H	83:17	75:25	1,75 (<1,50)	37 (<40)	733 (<820)

**F4 II-3**	75	Yes (50)	652C > T; 664G > T	P218S; V222L	81:19	82:18	1,57 (<0,75)	19 (<17)	1680 (<1100)

**F4 III-1**	44	No	652C > T; 664G > T	P218S; V222L	83:17	81:19	2,38 (<1,50)	53 (<40)	1424 (<820)

**F4 III-3**	45	Yes (29)	652C > T; 664G > T	P218S; V222L	89:11	82:18	1,00 (<0,75)	36 (<17)	1611 (<1100)

**F5 II-1**	55	Yes (54)	1202G > A	R401Q	98:02	82:18	1,96 (<1,50)	38 (<40)	1031 (<820)

**F6 II-1**	76	Yes (58)	1727T > C	L576P	73:27	76:24	2,10 (<0,75)	21 (<17)	1039 (<1100)

**F7 I-2**	72	No	1772G > A	R591Q	n/a	n/a	1,23 (<1,5)	16 (<40)	798 (<820)

**F7 II-1**	44	Yes (34)	1772G > A	R591Q	96:04	97:03	2,7 (<1,50)	56 (<40)	957 (<820)

**F8 II-1**	62	Yes (40)	1992G > A	W664X	83:17	82:18	3,08 (<1,50)	56 (<40)	1132 (<820)

**F9 II-1**	63	No	293C > T	S98L	83:17	93:07	1,82 (<1,50)	37 (<40)	888 (<820)

**F9 II-3**	57	No	293C > T	S98L	79:21	75:25	1,99 (<1,50)	42 (<40)	913 (<820)

**F9 III-2**	20	No	293C > T	S98L	75:25	61:39	2,65 (<1,50)	46 (<40)	1149 (<820)

**F10 I-2**	63	No	443A > G	N148S	86:14	42:58	2,16 (<1,50)	42 (<40)	788 (<820)

**F10 II-2**	40	No	443A > G	N148S	96:04	84:16	2,17 (<1,50)	43 (<40)	757 (<820)

**F11 III-1**	67	No	1165C > T	R389C	52:48	72:28	0,7 (<1,50)	13 (<40)	572 (<820)

**F11 III-3**	64	No	1165C > T	R389C	78:22	34:66	1,1 (<1,50)	16 (<40)	823 (<820)

**F11 III-5**	49	No	1165C > T	R389C	98:02	20:80	1,05 (<1,50)	16 (<40)	848 (<820)

**F11 III-6**	46	No	1165C > T	R389C	71:29	74:26	1,30 (<1,50)	18 (<40)	1000 (<820)

**F11 V-1**	26	No	1165C > T	R389C	57:43	58:42	0,68 (<1,50)	14 (<40)	663 (<820)

**F12 I-2**	53	No	1211C > A	S404X	95:05	09:91	nd	nd	nd

**F13 I-2**	60	No	del. ex8-10	n/a	76:24	nd	nd	nd	nd

**F13 II-2**	37	No	del. ex8-10	n/a	93:07	nd	1,99 (<1,50)	37 (<40)	1040 (<820)

**F13 II-3**	30	No	del. ex8-10	n/a	99:01	nd	nd	nd	nd

**F14 I-2**	52	No	del. ex7-10	n/a	69:31	nd	1,91 (<1,50)	34 (<40)	606 (<820)

del, deletion; n/a, not applicable; nv, normal value; nd, not done.

**Table 2:** Clinical findings of the symptomatic ALD female carriers

	F1 II-3	F2 I-2	F3 II-2	F4 II-3	F4 III-3	F5 II-1	F6 II-1	F7 II-1	F8 II-1
**Age at evaluation (yrs)**	67	61	73	75	45	55	76	44	62

**Age at onset**	45	59	45	50	29	54	58	34	40

**Symptoms at onset**	Difficulty to walk	Difficulty to walk and imbalance	Difficulty to walk	Difficulty to walk	Difficulty to walk & Back pain	Mild leg stiffness	Difficulty to walk	Difficulty to run	Difficulty to walk & Leg stiffness

**Gait at examination**	Paraparetic & ataxic gait	Paraparetic	Paraparetic	Paraparetic	Paraparetic	Near-normal	Paraparetic	Paraparetic	Paraparetic

**Ambulation Index**^**1**^	4	3	6	8	5	1	4	4	4

**Muscle tone (legs)**^**2**^	Increased (++)	Increased (+)	Increased (+++)	Increased (++)	Increased (+)	Increased (+)	Increased (+)	Increased (++)	Increased (++)

**Tendon reflexes**	Increased	Increased	Increased	Increased	Increased	Increased	Increased	Increased	Increased

**Plantar response**	Extensor	Extensor	Extensor	Extensor	Extensor	Extensor	Bilaterally indifferent	Extensor	Extensor

**Vibration sense (legs)**^**3**^	Impaired (++)	Impaired (++)	Impaired (++)	Impaired (++)	Impaired (+)	Impaired (++)	Impaired (+)	Impaired (++)	Impaired (++)

**Bladder function**	Urge-incontinence	Urge-incontinence	Urge-incontinence	Urge-incontinence	Urge-incontinence	Normal	Incontinence	Normal	Normal

**Other signs**	No	Lumbar hyperlordosis	Mild upper limb ataxia	No	No	Lumbar hyperlordosis	No	Lumbar hyperlordosis	Lumbar hyperlordosis

**Brain MRI**	Normal	Normal	Normal	n.d.	Normal	Normal	Cerebral atrophy	Normal	Normal

**MEPs**	n.d.	Abnormal	Abnormal	n.d.	n.d.	Near-normal	Abnormal	Abnormal	Abnormal

**SSEPs**	n.d.	Abnormal	Abnormal	n.d.	n.d.	Abnormal	Abnormal	Abnormal	n.d.

**VEPs**	n.d.	Abnormal	Normal	n.d.	n.d.	Abnormal	n.d.	Normal	n.d.

**BAEPs**	n.d.	Abnormal	Abnormal	n.d.	n.d.	Normal	n.d.	Abnormal	n.d.

**NCVs**	n.d.	Normal	Normal	n.d.	Abnormal	Normal	Abnormal	Abnormal	Normal

**Serum Cortisol**	Normal	n.d.	Normal	n.d.	n.d.	Normal	n.d.	Normal	n.d.

BAEPs, brainstem auditory evoked potentials, MEPs, motor evoked potentials; MRI, magnetic resonance imaging; n.d. = not done; NCVs, nerve conduction velocities; SSEPs, somato-sensory evoked potentials; VEPs, visual evoked potentials.

^1^For details, see Hauser *et al.*, 1983 [23].

^2 ^+ = mild; ++ = moderate; +++ = severe.

^3 ^+ = vibration sense decreased in feet; ++ = vibration sense absent in feet, and lowered in lower legs [24].

### Mutational Analysis and Biochemical Evaluation

After the informed consent was obtained, the genomic DNA of each female was extracted from blood samples collected in potassium EDTA tubes using standard procedures. The coding sequences (exons 1-10) and intron-exon boundaries of the *ABCD1 *gene were analyzed for mutations by direct sequence analysis using an automated system (ABI 3130xl). The primers used are available on request. The missense unknown variants were tested in 100 healthy Italian donors by direct sequencing. Major deletions in the *ABCD1 *gene were detected using the SALSA MLPA kit P049 SLC6A8 - ABCD1 (MRC-Holland). Note that deletions of probe recognition sequences in males are apparently due to the absence of the probe amplification product, whereas in heterozygous females, we detect a 35-50% reduction in the relative peak area of the amplification product of that probe. However, mutations and/or polymorphisms very close to the probe ligation site may also result in a reduced relative peak area. Therefore, apparent deletions detected by a single probe require confirmation by other methods (i.e., Real Time PCR, High Resolution Melting assay, or both). VLCFAs were quantified in plasma by standard procedures [10-12]

### X-Chromosome Inactivation (XCI) Assay and Pyrosequencing™ technology

The XCI pattern was assessed in DNA from white blood cells (WBCs) of 30 X-ALD carriers and 268 age-matched controls using methylation sensitive restriction enzymes, flanked by HUMARA (Androgen Receptor *locus*) and DXS6673E Single Tandem Repeats (STRs). PCR was performed on samples before and after enzymatic digestions, using the *HpaII *and *HhaI *enzymes (Boehringer Ingelheim, Mannheim, Germany) for HUMARA and *HhaI *and *RsaI *for DXS6673E as previously reported [13,14]. All samples were tested in duplicate and one male DNA sample was included in each experiment as a control for enzymatic digestion. PCR products were run by capillary electrophoresis and XCI values were determined in heterozygous cases using the formula previously reported [15]. The XCI pattern was defined as "moderately" skewed in the presence of an XCI ratio ≥75:25 and "severely" skewed when the XCI ratio was ≥90:10, similar to previous reports [15].

Our prior experience suggests that the skewing of XCI can be underestimated, as the cleavage of the unmethylated restriction sites can be incomplete due to partial efficiency of methyl-sensitive restriction enzymes. This phenomenon is "sample-dependent" (i.e. a single sample could be incompletely digested, even if the control male is fully digested), and may explain the high variability observed among different studies about the XCI pattern. To prevent this bias, we tested different restriction enzymes, and we used Pyrosequencing™ technology to detect the efficiency of the digestion (Additional file 2, Figure S2). Pyrosequencing allowed us to quantitatively evaluate the percentage of C/T (corresponding to methylated/unmethylated allele) in a sample. The digestion was considered complete when the percentage of residual unmethylated allele was below 5%. This procedure was performed in all samples and XCI assay was assessed only in samples showing a complete digestion.

### RNA extraction and Allele-Specific Expression (ASE) Assay

The RNAs were collected using Tempus Blood RNA tubes (Applied Biosystems) and isolated using the Tempus Spin RNA Isolation kit (Applied Biosystems), according to the manufacturer's procedures. cDNAs were synthesized using the High Capacity cDNA Reverse Transcription Kit (Applied Biosystems).

To assess the allele-specific expression (ASE), *ABCD1 *cDNA fragments containing the mutation were amplified, and PCR products were sequenced by the PyroMark ID instrument (Qiagen) following the manufacturer's instructions, and using specific PCR and sequencing primers (Additional file 3, Table S1). The contribution of the mutant and wild-type alleles were quantitatively measured by the Pyro Mark ID software v1.0.9 (Qiagen, Chatsworth, CA), which gives the percentage ratio of each allele. The quantification is highly precise with an error estimated as ≤10%. For this reason, we considered that one allele was higher expressed than the other, when their ratio was equal or higher than 1.5 (60:40). For each sample, allele-specific expression value represents the mean of at least two independent PCR and pyrosequencing experiments.

### Statistical Analyses

Fisher's exact test, Mann-Whitney Test and Spearman correlation were performed using GraphPad Prism version 5.04 for Windows, GraphPad Software, San Diego California USA.

## Results

### Clinical findings

We investigated 22 asymptomatic and 9 symptomatic ALD female carriers whose clinical, genetic and biochemical findings are summarized in Table 1. The clinical features of symptomatic women were similar to those previously reported, mainly characterized by spastic paraplegia, lower limb sensory disturbances, and neurogenic bladder (Table 2) [3,9]. The age at evaluation was significantly higher in the symptomatic than in asymptomatic females (62.0 ± 12.1 *vs*. 47.5 ± 14.1years; p = 0.0199, Mann-Whitney test). However, we did not find any significant difference between the age at onset of clinical symptoms in symptomatic carriers and the age at evaluation in the asymptomatic ones (46.0 ± 10.4 *vs*. 47.5 ± 14.1 years; p = 0.7276, Mann-Whitney test).

### Mutational analysis

The *ABCD1 *mutations of the female carriers are summarized in Table 1. We found 15 different *ABCD1 *mutations, five of which were new, according to the X-linked Adrenoleukodystrophy Database (http://www.x-ald.nl/). Additional information about the position on *ABCD1 *gene are available in Additional file 4, Table S2. As one carrier (F7 I-2) was a mosaic for the c.1772G > A mutation, she was excluded from the statistical analyses.

### X Chromosome Inactivation pattern

**Figure 1:**
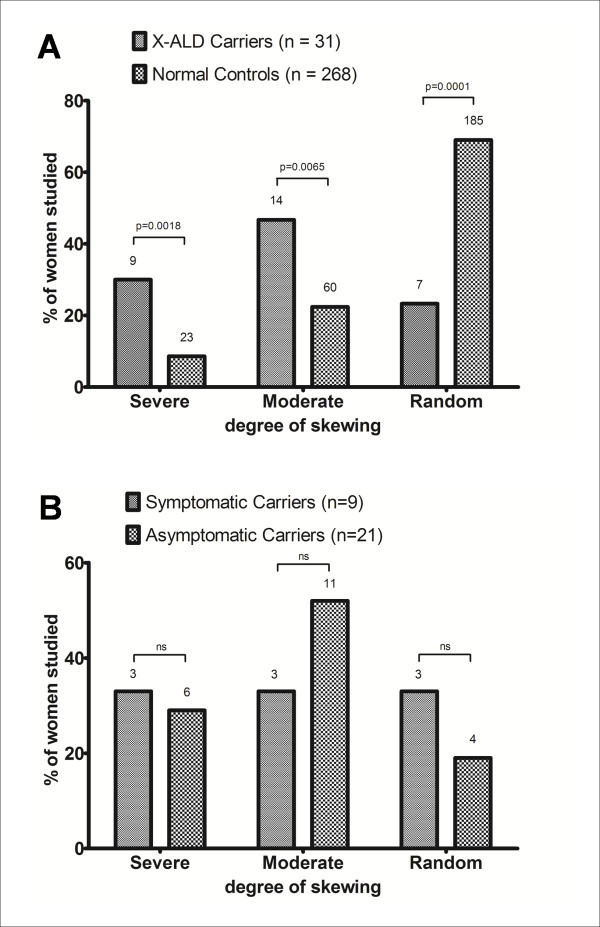
**Distribution of the X chromosome inactivation pattern in peripheral blood mononuclear cells of X-ALD female carriers and healthy controls**. A) A severe (≥90:10) skewing of XCI was found in 30% of X-ALD carriers (9/30) and 8.6% (23/268) of healthy controls (HCs) (p = 0.0018, Fisher's exact test); a moderate (≥75:25) skewing of XCI was found in 46.7% of carriers (14/30) and 22.4% (60/268) of HCs (p = 0.0065, Fisher's exact test); a random XCI was found in 23.3% of carriers (7/30) and 69% (185/268) of HCs (p = 0.0001, Fisher's exact test). B) A severe skewing of XCI was found in 3/9 symptomatic and 6/21 asymptomatic carriers (p = 1.00; Fisher's exact test); a moderate skewing of XCI was found in 3/9 symptomatic and 11/21 asymptomatic carriers (p = 0.4397; Fisher's exact test); a random XCI was found in 3/9 symptomatic and 4/21 asymptomatic carriers(p = 0.6402; Fisher's exact test). Severe = ≥90:10; moderate = 75:25 to 89:11; random = 50:50 to 74:26.

XCI analysis was performed in 30 X-ALD carriers and in 268 age-matched healthy females. As shown in Table 1, we found severely (≥90:10) skewed XCI in 30% (9/30) of X-ALD carriers and in 8.6% (23/268) controls; moderately (≥75:25) skewed XCI was found in 46.7% (14/30) carriers and in 22.4% (60/268) controls; random XCI in 23.3% (7/30) carriers and in 69% (185/268) controls. These findings indicate that the occurrence of both severe and moderate XCI skewing is significantly higher in X-ALD carriers *vs*. female controls (p = 0.0018 and p = 0.0065; Fisher's exact test - Table 1; Figure 1A). In contrast, we did not observe differences in XCI pattern between symptomatic and asymptomatic carriers (p = 0.6472; Mann-Whitney test - Table 1; Figure 1B). Indeed, we found severely skewed XCI in 3/9 symptomatic and 6/21 asymptomatic carriers (p = 1.00; Fisher's exact test); moderately skewed XCI in 3/9 symptomatic and 11/21 asymptomatic carriers (p = 0.4397; Fisher's exact test); random XCI in 3/9 symptomatic and 4/21 asymptomatic carriers (p = 0.6402; Fisher's exact test). The increase of age has been associated, by other studies [16,17], with a more frequent occurrence of preferential XCI but, in our population, no correlation was found between the degree of XCI of the X-ALD carriers and the age at evaluation (p = 0.1550; Spearman ρ correlation). This suggests that *ABCD1 *mutations may influence the XCI process, thus making the possible correlation between older age and preferential XCI irrelevant.

The overall results indicate that in X-ALD carriers the skewed XCI in PBMCs is highly frequent and not related to the manifestation of symptoms.

### Allele-specific expression of ABCD1 in X-ALD carriers

**Figure 2:**
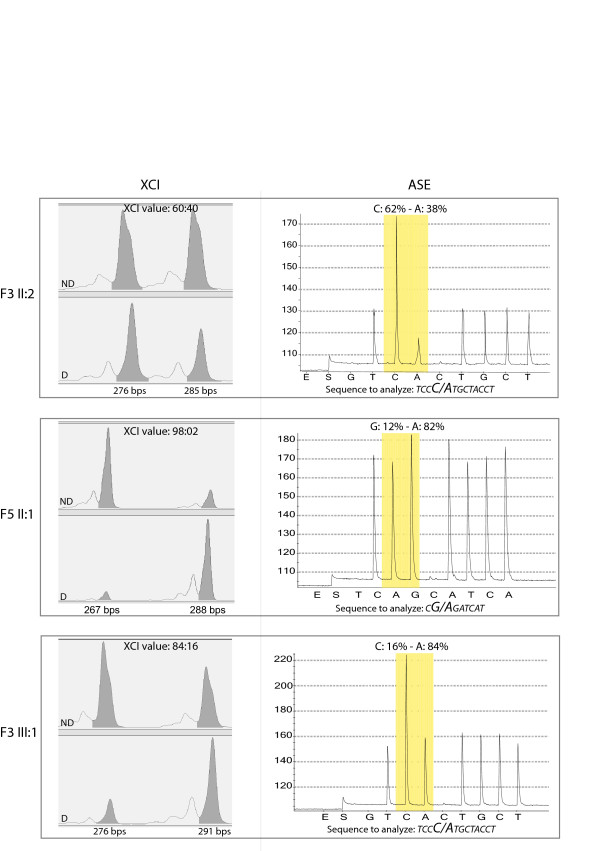
**X Chromosome Inactivation (XCI) and *ABCD1 *Allele-Specific Expression (ASE) results in three representative samples**. In the left column the electropherograms of HUMARA alleles ND (upper) and D (lower) are depicted. For each sample, the peak lengths (bps) and the corresponding area are indicated. The XCI results (ratio) are reported. In the right column the pyrograms of ASE experiments are shown and the analysed sequences of cDNA, containing the mutations (the base-change is highlighted in bold), are indicated. The mutation site is boxed in grey and the ASE (percentage of wt: mutant allele) is indicated. Sample F3 II-2shows a random XCI pattern and only a slight imbalance in *ABCD1 *ASE, suggesting that *ABCD1 *follows XCI; sample F5 II-1is characterized by severe skewing of both XCI and *ABCD1 *ASE, with an evident preferential expression of the mutant allele; in F3 III-1 sample XCI is moderately skewed, as well as the *ABCD1 *ASE. ND = DNA not digested D = DNA digested.

*ABCD1 *Allele-Specific Expression (ASE) was evaluated in 25 X-ALD females carrying point mutations, as cases showing deletions or mosaicisms were not suitable for this experimental approach. As shown in Table 1, overall, we found a predominant expression (>60%) of one allele with respect to the other in 23 out of the 25 carriers. Specifically, the mutant allele was more expressed (range: 61% - 97%) than the wild-type in 19 out of 23 carriers and less expressed (range: 9% - 38%) in four out of 23 carriers. Importantly, among the 23 carriers with a predominant expression of one allele, we did not observe any difference in ASE between the symptomatic (n = 9) and the asymptomatic (n = 14) X-ALD carriers (p = 0.0938; Mann-Whitney test). Indeed, the mutant allele was predominantly expressed in 8 out of 9 symptomatic X-ALD carriers, and in 11 out of 14 asymptomatic carriers (p = 1.00; Fisher's exact test). Therefore, it is not surprising that 63 year-old patient F9 II:1 was asymptomatic, despite the predominant (93%) expression of the mutant *ABCD1 *allele, while 73 year-old patient F3 II:2 was symptomatic, despite the predominant (62%) expression of the wild-type *ABCD1 *allele. We also found that the *ABCD1 *ASE pattern usually mirrors the XCI pattern (i.e., their difference is <10%), probably because one allele *of ABCD1 *is usually silenced as result of the XCI. Representative results are shown in Figure 2. Accordingly, we observed a significant correlation between the ASE and XCI pattern (p < 0.0001, Mann-Whitney test). Overall, our ASE data show that the skewing of XCI toward the mutant *ABCD1 *allele is common but not constant, and that the degree of expression of the mutant *ABCD1 *allele is not related to the presence of symptoms.

### Biochemical characterization

In our series plasma concentration of C26:0, the ratio C26:0 to C22:0, and the ratio C24:0 to C22:0 were all in the normal range in about 15% of female carriers. Interestingly, in 17 out of 22 assessable X-ALD carriers (Table 1), we observed a significant positive correlation between the degree of mutant *ABCD1 *expression and the plasma concentration of C26:0 (p = 0.0201; Spearman ρ correlation) or the ratio C26:0 to C22:0 (p = 0.0141). This evidence demonstrates *in vivo *a previous suggestion obtained by *in vitro *studies [18]. We note that the remaining five out of 22 assessable carriers were excluded from the statistical analysis, as the blood for the VLCFA quantification was sampled a few years before that for RNA extraction, and a different method for their biochemical evaluation was used (Table 1).

## Discussion

In this study we assessed the pattern of both X Chromosome Inactivation (XCI) and *ABCD1 *allele-specific expression (ASE) in the peripheral blood mononuclear cells (PBMCs) of 30 female X-ALD carriers.

First, we observed, by a strictly controlled and very reproducible experimental procedure, that the skewing of XCI is significantly more common in X-ALD carriers than in healthy females, in agreement with previous findings [8].

Second, by ASE analyses, we provided the first evidence that, in X-ALD carriers, the active X chromosome is typically the chromosome carrying the mutant *ABCD1 *allele.

It is already known that the severity of clinical symptoms in X-linked disorders can be influenced by XCI skewing [19]. Our finding of a skewed XCI favoring the inactivation of the wild-type allele is peculiar. Indeed, the skewing of XCI seems to commonly favor the inactivation of the mutant allele, thus explaining why, in many severe X-linked disorders, female carriers may be asymptomatic and have a severely skewed XCI [5,6]. This is the case of Lesch-Nyhan syndrome, in which the cells expressing the wild-type allele of *HPRT1 *reproduce faster, thus gradually causing decrease of the cells in which the mutant allele is expressed [20]. Similarly, in Wiskott-Aldrich syndrome, female carriers show preferential inactivation of the mutant allele in hematopoietic cells [20]. In contrast, X-ALD had already been considered as the example of X-linked disease in which *ABCD1 *mutations confer a proliferative advantage rather than disadvantage, leading to XCI skewing in favor of the X chromosome with the mutation [5,6,8,18], an evidence here demonstrated *in vivo*. This type of skewing could be due to a somatic selection in favor of cells carrying an active mutated X chromosome, after the X-inactivation process; in turn, this positive selection might be due to a growth advantage of cells expressing the defective *ABCD1 *allele [18]. However, it remains enigmatic why cells expressing a mutant *ABCD1 *should have a growth advantage over normal cells. Reduced intracellular VLCFA β-oxidation, due to the mutant *ABCD1*, and the consequent change of lipid environment might be the basis for this phenomenon.

Moreover, we have investigated whether both the skewing of XCI or the preferential expression of the mutant *ABCD1 *allele were related to the neurological manifestations affecting a subgroup of female carriers. Our data do not support any significant correlation between neurological manifestations of X-ALD carriers and the XCI pattern or the degree of expression of the mutant *ABCD1 *allele. The lack of correlation between neurological manifestations and XCI pattern in PBMCs is in agreement with previous data of Watkiss *et al *and Jung *et al *[7,21], and in contrast with those of Maier *et al *[8]. Since XCI pattern evaluation could be biased by experimental procedures, it is conceivable that discrepancies between our results and previous results are due to the different methodologies used. Indeed, our data were obtained in a highly reproducible manner and in a cohort of X-ALD female carriers larger than those previously studied. The lack of correlation between neurological manifestations and XCI pattern or degree of expression of the mutant *ABCD1 *allele might be due to the tissue analyzed, as the degree of XCI or mutant *ABCD1 *expression might be different in the brain, which is the tissue mainly involved in X-ALD. Although XCI in brain from X-ALD carriers was not investigated, a similar XCI pattern in blood and brain has been observed [22]. To explore the maintenance of XCI pattern in another tissue we performed XCI and ASE in urinary sediment of a small subset of our patients (n = 8) and we have found that XCI and ASE pattern were similar in the two cell types (data not shown).

Finally, our data reveal a significant and causative correlation between the VCLFA plasma concentrations and the degree of mutant *ABCD1 *allele-specific expression *in vivo*. This is in agreement with previous *in vitro *observations by Migeon *et al *[18].

In conclusion, we provide evidence that: i) in X-ALD carriers the high frequency of non-random XCI usually reflects the preferential expression of the mutant *ABCD1 *allele, thus supporting by *in vivo *data the hypothesis relating to the growth advantage of cells expressing the mutant *ABCD1 *[18]; ii) the quantitative allele-specific expression mirrors the plasma concentration of VCLFAs; iii) unfortunately, due to the lack of correlation between XCI/ASE pattern and clinical symptoms, our results prove that these molecular values cannot be considered in clinical practice as predictive markers for the development of symptoms in X-ALD carriers.

## Authors' contributions

ES and ST are the principal investigators of the study: they planned the experimental tasks and the statistical analysis plan; ES also recruited the subjects for the study. SMS designed and implemented data collection and revised the draft paper; PC designed and implemented the experiments; BC analyzed and monitored the data; CG monitored data collection and performed statistical analyses; MR implemented the data and contributed to statistical analysis; VP collected and analyzed the data; CM recruited the subjects and revised data analyses; DP participated to experimental plan and drafted and revised the paper; MM coordinated the experimental plan and analyzed the data. She critically revised the manuscript and the final approval of the version to be published. GU initiated the collaborative project, recruited the subjects for the study, obtained funding for the study, drafted and revised the paper for intellectual content; she is guarantor. All authors read and approved the final manuscript.

## Supplementary Material

Additional file 1 **Pedigrees of 14 families in which X-ALD disease segregates**. Click here for file

Additional file 2 **HUMARA assay validation by Pyrosequencing**. **A **- The "HUMARA assay validation by Pyrosequencing" was designed to verify the efficiency of the enzymatic digestions of samples. Hpa II and Hha I restriction enzymes cut DNA at multiple sites of the Androgen Receptor locus. Notably, they recognize and digest only the unmethylated strand, whereas the methylated one remains undigested. After enzymatic restriction, DNA was bisulphite converted, allowing the conversion of the unmethylated "C" in "T" and PCR reaction was performed on the converted DNA. Since the unmethylated DNA has been cut by restriction enzymes, its amplification is virtually impossible. Pyrosequencing analysis is able to detect the presence of PCR products from the unmethylated strand (in which, after bisulphate conversion, all "C" have switched to "T"), by the retrieval of residual "T" on the final sequence. **B **- Example of representative pyrograms of the bisulphite-converted DNA sample F10 III-3- PCR products: 1) undigested, 2) showing incomplete or 3) complete digestion, using different experimental conditions. Click here for file

Additional file 3 **PCR and pyrosequencing primer sequences, together with the sequences analyzed by the PyroMarkID instrument in ASE analyses**. Click here for file

Additional file 4 ***ABCD1 *gene mutations in female carriers cohort**. The *ABCD1 *mutations are arranged based on the nucleotide position. According to the X-linked Adrenoleukodsytrophy database (http://www.x-ald.nl/), the amino acid substitution, the exon in which the mutation occurs, and the protein status in fibroblasts are indicated. Note that two new mutations (c.652C > T and c.664G > T) were found to be in *cis *on the same allele. ALDP = AdrenoLeukoDystrophy Protein; N/A = not applicable; n.d. = no data provided; * = new mutation. Click here for file

## References

[B1] Fourcade S López-Erauskin J Galino J Duval C Naudi A Jove M Kemp S Villarroya F Ferrer I Pamplona R Portero-Otin M Pujol A Early oxidative damage underlying neurodegeneration in X-adrenoleukodystrophy Hum Mol Genet 2008 17 1762 1773 10.1093/hmg/ddn085 18344354

[B2] Moser HW Loes DJ Melhem ER Raymond GV Bezman L Cox CS Lu SE X-Linked adrenoleukodystrophy: overview and prognosis as a function of age and brain magnetic resonance imaging abnormality. A study involving 372 patients Neuropediatrics 2000 31 227 239 10.1055/s-2000-9236 11204280

[B3] Schmidt S Träber F Block W Keller E Pohl C von Oertzen J Schild H Schlegel U Klockgether T Phenotype assignment in symptomatic female carriers of X-linked adrenoleukodystrophy J Neurol 2001 248 36 44 10.1007/s004150170267 11266018

[B4] Dobyns WB Filauro A Tomson BN Chan AS Ho AW Ting NT Oosterwijk JC Ober C Inheritance of most X-linked traits is not dominant or recessive, just X-linked Am J Med Genet A 2004 129A 136 143 10.1002/ajmg.a.30123 15316978

[B5] Van den Veyver IB Skewed X inactivation in X-linked disorders Semin Reprod Med 2001 19 183 191 10.1055/s-2001-15398 11480916

[B6] Ørstavik KH X chromosome inactivation in clinical practice Hum Genet 2009 126 363 373 10.1007/s00439-009-0670-5 19396465

[B7] Watkiss E Webb T Bundey S Is skewed X inactivation responsible for symptoms in female carriers for adrenoleucodystrophy? J Med Genet 1993 30 651 654 10.1136/jmg.30.8.651 8411051 PMC1016492

[B8] Maier EM Kammerer S Muntau AC Wichers M Braun A Roscher AA Symptoms in carriers of adrenoleukodystrophy relate to skewed X inactivation Ann Neurol 2002 52 683 688 10.1002/ana.10376 12402273

[B9] Semmler A Köhler W Jung HH Weller M Linnebank M Therapy of X-linked adrenoleukodystrophy Expert Rev Neurother 2008 8 1367 1379 10.1586/14737175.8.9.1367 18759549

[B10] Uziel G Bertini E Bardelli P Rimoldi M Gambetti M Experience on therapy of adrenoleukodystrophy and adrenomyeloneuropathy Dev Neurosci 1991 13 274 279 10.1159/000112173 1817033

[B11] Johnson DW Alkyldimethylaminoethyl ester iodides for improved analysis of fatty acids by electrospray ionization tandem mass spectrometry Rapid Commun Mass Spectrom 2000 14 2019 2024 10.1002/1097-0231(20001115)14:21<2019::AID-RCM121>3.0.CO;2-2 11085412

[B12] Johnson DW A rapid screening procedure for the diagnosis of peroxisomal disorders: quantification of very long-chain fatty acids, as dimethylaminoethyl esters, in plasma and blood spots, by electrospray tandem mass spectrometry J Inherit Metab Dis 2000 23 475 486 10.1023/A:1005612214179 10947202

[B13] Allen RC Zoghbi HY Moseley AB Rosenblatt HM Belmont JW Methylation of HpaII and HhaI sites near the polymorphic CAG repeat in the human androgen-receptor gene correlates with X chromosome inactivation Am J Hum Genet 1992 51 1229 1239 1281384 PMC1682906

[B14] Beever C Lai BP Baldry SE Peñaherrera MS Jiang R Robinson WP Brown CJ Methylation of ZNF261 as an assay for determining X chromosome inactivation patterns Am J Med Genet 2003 120A 439 441 10.1002/ajmg.a.20045 12838571

[B15] Miozzo M Selmi C Gentilin B Grati FR Sirchia S Oertelt S Zuin M Gershwin ME Podda M Invernizzi P Preferential X chromosome loss but random inactivation characterize primary biliary cirrhosis Hepatology 2007 46 456 462 10.1002/hep.21696 17659578

[B16] Hatakeyama C Anderson CL Beever CL Peñaherrera MS Brown CJ Robinson WP The dynamics of X-inactivation skewing as women age Clin Genet 2004 66 327 332 10.1111/j.1399-0004.2004.00310.x 15355435

[B17] Busque L Paquette Y Provost S Roy DC Levine RL Mollica L Gilliland DG Skewing of X-inactivation ratios in blood cells of aging women is confirmed by independent methodologies Blood 2009 113 3472 3474 10.1182/blood-2008-12-195677 19202126 PMC4729536

[B18] Migeon BR Moser HW Moser AB Axelman J Sillence D Norum RA Adrenoleukodystrophy: evidence for X linkage, inactivation, and selection favoring the mutant allele in heterozygous cells Proc Natl Acad Sci USA 1981 78 5066 5070 10.1073/pnas.78.8.5066 6795626 PMC320333

[B19] Migeon BR Non-random X chromosome inactivation in mammalian cells Cytogenet Cell Genet 1998 80 142 148 10.1159/000014971 9678349

[B20] Migeon BR The role of X inactivation and cellular mosaicism in women's health and sex-specific diseases JAMA 2006 295 1428 1433 10.1001/jama.295.12.1428 16551715

[B21] Jung HH Wimplinger I Jung S Landau K Gal A Heppner FL Phenotypes of female adrenoleukodystrophy Neurology 2007 68 960 961 10.1212/01.wnl.0000257129.51273.73 17372139

[B22] Bittel DC Theodoro MF Kibiryeva N Fischer W Talebizadeh Z Butler MG Comparison of X-chromosome inactivation patterns in multiple tissues from human females J Med Genet 2008 45 309 313 10.1136/jmg.2007.055244 18156436 PMC5489244

[B23] Hauser SL Dawson DM Lehrich JR Beal MF Kevy SV Propper RD Mills JA Weiner HL Intensive immunosuppression in progressive multiple sclerosis. A randomized, three-arm study of high-dose intravenous cyclophosphamide, plasmaexchange, and ACTH N Engl J Med 1983 308 173 180 10.1056/NEJM198301273080401 6294517

[B24] van Geel BM Koelman JH Barth PG Ongerboer de Visser BW Peripheral nerve abnormalities in adrenomyeloneuropathy: A clinical and electrodiagnostic study Neurology 1996 46 112 118 8559356 10.1212/wnl.46.1.112

