# Supplementary figures and images for: Preferential expression of mutant ABCD1 allele is common in adrenoleukodystrophy female carriers but unrelated to clinical symptoms
Source: Orphanet J Rare Dis. 2012 Jan 26;7:10. doi: 10.1186/1750-1172-7-10 (PMC3298485; doi:10.1186/1750-1172-7-10)

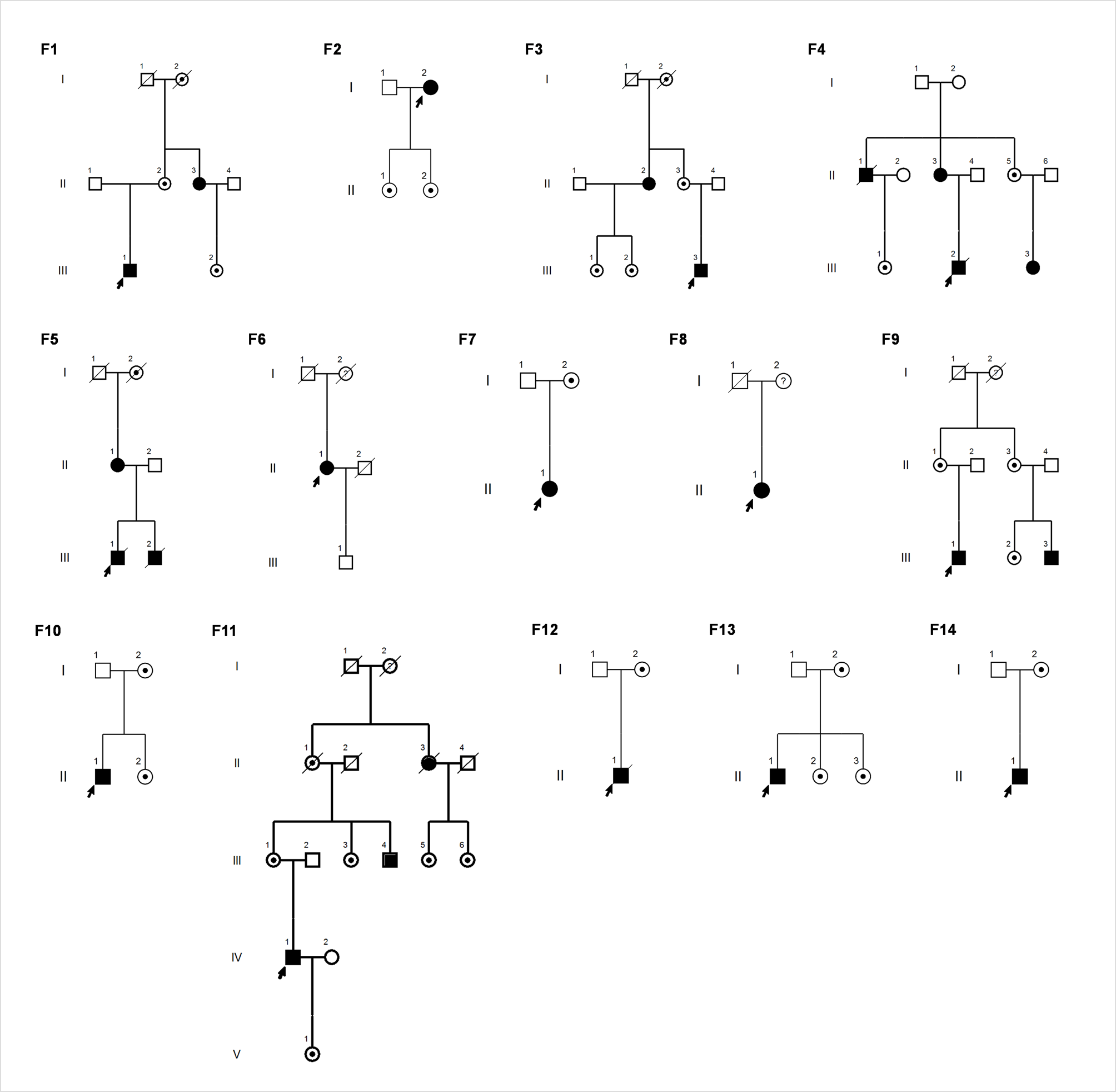

Supplement: Additional file 1 — Pedigrees of 14 families in which X-ALD disease segregates. [file 1750-1172-7-10-S1.TIFF]
