# Supplementary material for: Preferential expression of mutant ABCD1 allele is common in adrenoleukodystrophy female carriers but unrelated to clinical symptoms
Source: Orphanet J Rare Dis. 2012 Jan 26;7:10. doi: 10.1186/1750-1172-7-10 (PMC3298485; doi:10.1186/1750-1172-7-10)
Supplement: Additional file 3 — PCR and pyrosequencing primer sequences, together with the sequences analyzed by the PyroMarkID instrument in ASE analyses. [file 1750-1172-7-10-S3.DOC]

| **Mutation** | **PCR (F+R) and Pyrosequencing (S) primers (5' --> 3)'** | | **PCR product lenght (bps)** | **Sequence analyzed (5' ---> 3')** |
| --- | --- | --- | --- | --- |
|  |  |  |  |  |
| **C293T** | F | CAGCGGCTCCTGTGGCTC | 253 | ***C/T****GGCCGCCTTG* |
|  | R* | GGCCCTCCAGGTAACGGAT |  |  |
|  | S | TGCTGGCCCTGCACT |  |  |
|  |  |  |  |  |
| **G410A** | F | CAGCGGCTCCTGTGGCTC | 253 | *T****G/A****GCTCCTC* |
|  | R* | GGCCCTCCAGGTAACGGAT |  |  |
|  | S | CTGGCAGCTGCTGCAG |  |  |
|  |  |  |  |  |
| **C427G** | F | TGCAGTGGCTCCTCATCG | 66 | *TC****C/G****CTGCTACCTTCG* |
| **and** | R* | GGCCCTCCAGGTAACGGAT |  | *and* |
| **C428A** | S | GGCTCCTCATCGCCC |  | *TCCC/ATGCTACCT* |
|  |  |  |  |  |
| **A443G** | F | TGCAGTGGCTCCTCATCG | 66 | ***A/G****CAGTGCC* |
|  | R* | GGCCCTCCAGGTAACGGAT |  |  |
|  | S | CCCTGCTACCTTCGTCA |  |  |
|  |  |  |  |  |
| **C652T** | F | TGGCCCACCTCTACTCCAAC | 345 | ***C/T****CACTCCTGGAC****G/T****TGG* |
| **and** | R* | CCAGAAGGATGAGGTTGATCT |  |  |
| **G664T** | S | CTCCAACCTGACCAAG |  |  |
|  |  |  |  |  |
| **C1165T** | F | CTACTCAGAGTCAGATGCAGA | 171 | ***C/T****GCAACCTC* |
|  | R* | GCCAGCTCCGTCACCTCCT |  |  |
|  | S | AGCCTTCACTATTGCC |  |  |
|  |  |  |  |  |
| **G1202A** | F | CTACTCAGAGTCAGATGCAGA | 171 | *C****G/A****GATCAT* |
| **and** | R* | GCCAGCTCCGTCACCTCCT |  | *and* |
| **C1211A** | S | GCTGCAGATGCCATTGAG |  | *CGGATCATGTC/AGTCG* |
|  |  |  |  |  |
| **T1727C** | F | TACCCGGACTCAGTGGAGGA | 124 | *C****T/C****GGAAGCCATCCTG* |
|  | R* | CACACATAGCCTCCCAACCTC |  |  |
|  | S | GGCTACTCGGAGCAGGAC |  |  |
|  |  |  |  |  |
| **G1772A** | F | TACCCGGACTCAGTGGAGGA | 124 | ***G/A****GGAGGGAGGTTG* |
|  | R* | CACACATAGCCTCCCAACCTC |  |  |
|  | S | ACCACATCCTGCAGC |  |  |
|  |  |  |  |  |
| **G1992A** | F* | CATGTTCTACCACAGGCCC | 199 | ***C/T****CACAGGGAG* |
|  | R | CTTCTCGAACTTCCAGCCG |  |  |
|  | S | CAAGTGTGTGTGGTATTT |  |  |
|  | * Biotinilated primer | |  |  |

**Table S1**
