# Supplementary material for: Preferential expression of mutant ABCD1 allele is common in adrenoleukodystrophy female carriers but unrelated to clinical symptoms
Source: Orphanet J Rare Dis. 2012 Jan 26;7:10. doi: 10.1186/1750-1172-7-10 (PMC3298485; doi:10.1186/1750-1172-7-10)
Supplement: Additional file 4 — ABCD1 gene mutations in female carriers cohort. The ABCD1 mutations are arranged based on the nucleotide position. According to the X-linked Adrenoleukodsytrophy database (http://www.x-ald.nl/), the amino acid substitution, the exon in which the mutation occurs, and the protein status in fibroblasts are indicated. Note that two new mutations (c.652C > T and c.664G > T) were found to be in cis on the same allele. ALDP = AdrenoLeukoDystrophy Protein; N/A = not applicable; n.d. = no data provided; * = new mutation. [file 1750-1172-7-10-S4.DOC]

**Table S2**

| Mutation | Amino acids | Exon | ALDP |
| --- | --- | --- | --- |
| c.293C>T | p.S98L (Ser98Leu) | 1 | present |
| c.410G>A | p.W137X (Trp137X) | 1 | n.d. |
| c.427C>G (*) | p.P143A (Pro143Ala) | 1 | n.d. |
| c.428C>A | p.P143H (Pro143His) | 1 | present |
| c.443A>G | p.N148S (Asn148Ser) | 1 | present |
| c.652C>T (*) | p.P218S (Pro218Ser) | 1 | n.d. |
| c.664G>T (*) | p.V222L (Val222Leu) | 1 | n.d. |
| c.1165C>T | p.R389C (Arg389Cys) | 3 | n.d. |
| c.1202G>A | p.R401Q (Arg401Gln) | 3 | present |
| c.1211C>A (*) | p.S404X (Ser404X) | 3 | n.d. |
| c.1727T>C | p.L576P (Leu576Pro) | 7 | n.d. |
| c.1772G>A | p.R591Q (Arg591Gln) | 7 | present |
| c.1992G>A (*) | p.W664X (Trp664X) | 9 | n.d. |
| exon7-10del | N/A | - | absent |
| exon8-10del | N/A | - | n.d. |
